# Nutrient Regulation by Continuous Feeding Removes Limitations on Cell Yield in the Large-Scale Expansion of Mammalian Cell Spheroids

**DOI:** 10.1371/journal.pone.0076611

**Published:** 2013-10-18

**Authors:** Bradley P. Weegman, Peter Nash, Alexandra L. Carlson, Kristin J. Voltzke, Zhaohui Geng, Marjan Jahani, Benjamin B. Becker, Klearchos K. Papas, Meri T. Firpo

**Affiliations:** 1 Stem Cell Institute, Division of Endocrinology, Department of Medicine, University of Minnesota, Minneapolis, Minnesota, United States of America; 2 Schulze Diabetes Institute, Department of Surgery, University of Minnesota, Minneapolis, Minnesota, United States of America; 3 Institute for Cellular Transplantation, Department of Surgery, University of Arizona, Tucson, Arizona, United States of America; Wake Forest Institute for Regenerative Medicine, United States of America

## Abstract

Cellular therapies are emerging as a standard approach for the treatment of several diseases. However, realizing the promise of cellular therapies across the full range of treatable disorders will require large-scale, controlled, reproducible culture methods. Bioreactor systems offer the scale-up and monitoring needed, but standard stirred bioreactor cultures do not allow for the real-time regulation of key nutrients in the medium. In this study, β-TC6 insulinoma cells were aggregated and cultured for 3 weeks as a model of manufacturing a mammalian cell product. Cell expansion rates and medium nutrient levels were compared in static, stirred suspension bioreactors (SSB), and continuously fed (CF) SSB. While SSB cultures facilitated increased culture volumes, no increase in cell yields were observed, partly due to limitations in key nutrients, which were consumed by the cultures between feedings, such as glucose. Even when glucose levels were increased to prevent depletion between feedings, dramatic fluctuations in glucose levels were observed. Continuous feeding eliminated fluctuations and improved cell expansion when compared with both static and SSB culture methods. Further improvements in growth rates were observed after adjusting the feed rate based on calculated nutrient depletion, which maintained physiological glucose levels for the duration of the expansion. Adjusting the feed rate in a continuous medium replacement system can maintain the consistent nutrient levels required for the large-scale application of many cell products. Continuously fed bioreactor systems combined with nutrient regulation can be used to improve the yield and reproducibility of mammalian cells for biological products and cellular therapies and will facilitate the translation of cell culture from the research lab to clinical applications.

## Introduction

Cell replacement therapies in humans require the production of large-scale culture of viable, functioning cells. Reproducibility of cell product, and optimal cell yield and function all depend on the presence of appropriate levels of key nutrients, and sub-toxic levels of cell waste products [1], [2]. For research purposes, mammalian cells are typically cultured in static culture and propagated by passaging at regular intervals, with supplemental medium changes as needed. This method is limited by the requirement for frequent manipulations, which results in variability of culture conditions and increased risk of contamination [3]–[7]. Further, these culture methods are time intensive and require trained technicians to maintain large-scale cultures. Stirred suspension bioreactors (SSB) can be used as an alternative to static cell culture for microorganism cultures to increase culture volume and density, and decrease handling [8]. This approach has been applied to mammalian cells, including pluripotent stem cells [9]–[18]. However, SSB cultures still require interventions for medium changes, exhibit fluctuations in nutrient and waste product levels, and provide limited information about culture status. A perfusion system can be used to address these challenges by continuous infusion and removal of medium, but parameters such as calculating feed rate based on real-time cell requirements must be established [19]–[22].

In this study, SSB culture was used to expand an insulinoma cell line with many beta cell features intact, β-TC6 cells [23]–[27], to increase culture scale and improve cell expansion rates without compromising viability. These cells, like most mammalian cells, are dependent on a key nutrient, glucose, for energy production [28]. In addition, beta cells are sensitive to chronic high levels of glucose [29]. For this study, β-TC6 cells were allowed to form spheroids in culture approximating islet cluster sizes in vivo, and then allocated to either static or SSB culture conditions. While stirred bioreactors allowed the increase of culture volume by more than 10-fold, a continuous feeding perfusion bioreactor system [16]–[19], [30] was required to both maintain stable culture conditions, and maintain cell growth.

## Materials and Methods

### Cell Line and Maintenance

The β-TC6 cells were provided by the ATCC (Manassas, VA). In preparation for the study, they were cultured, passaged, and cryopreserved according to provider instructions in Dulbecco’s Modified Eagles Medium (DMEM, Invitrogen, Carlsbad, CA), with 4 mM L-glutamine, 4.5 g/L glucose and 1 mM sodium pyruvate (all from Invitrogen). Cells were passaged at a ratio of 1∶3 every 3–4 days.

### β-TC6 Spheroid Formation

This technique is described in literature [16]–[19], [31]–[33], and was slightly modified to accommodate spheroid formation of β-TC6 cells. For all conditions, β-TC6 cells were first cultured and expanded in adherent cultures described above, until enough cells were obtained to reach the required (total n = 12) numbers for 250 ml stirred bioreactors (Corning, Corning, NY). The cells were collected by gentle trypsinization (0.25% (w/v) Trypsin- 0.53 mM EDTA, Invitrogen) at room temperature aided by mechanical agitation for 2–3 minutes, and seeded into bioreactors at a density of 1.32×10^6^±5.7% cells/mL in 200 ml culture medium. Cells were then cultured in the bioreactors without feeding for 3 days at 37°C, with 5% CO_2_, 100% relative humidity, and stir rate of 70 rpm to allow spheroids to form. No significant proliferation was observed during the three day spheroid formation period. After spheroid formation, each bioreactor was allocated to a specific culture condition.

### Experimental Culture Conditions

After spheroid formation, spheroids were divided among three culture methods: static culture, stirred suspension bioreactor (SSB) culture, and continuously fed SSB culture. Cultures were compared at three different glucose concentrations (1.0 g/L, 2.75 g/L, and 4.5 g/L) to represent the range between physiological glucose (approximately 0.7 g/L) and standard β-TC6 culture medium (4.5 g/L). **Static Culture:** Spheroids from the initial bioreactor cultures were transferred to 10 cm diameter cell culture dishes (Nunc, Rochester, NY) containing 10 ml of culture medium. Parallel cultures were established for each glucose concentration; 100% medium exchange was done every 3 days by collecting the spheroids in 50 ml conical tubes (Falcon, San Jose, CA). The spheroids were gently pelleted using a refrigerated centrifuge at 4°C and 52.1×G. Culture medium was aspirated and pellets were re-suspended in fresh medium and placed back into culture. **Stirred Suspension Bioreactor Culture (SSB):** Stirred bioreactors remained in bioreactor conditions identical to spheroid formation cultures for the duration of the 21-day culture. Medium was replenished by performing a 100% medium exchange every three days. For media changes, cultures were removed from the bioreactor and processed as for static cultures above. Fresh medium was then added to re-establish the 200 ml culture volume. **Continuously Fed SSB Culture:** Stirred bioreactors identical to the SSB condition were adapted with a lid designed for this study to provide connection to a continuous feed. The feeding system, described in detail below, was designed to maintain stable culture conditions by continuously adding fresh medium at a regulated rate, and removing waste medium at the same rate. Initially, continuously fed SSB cultures had the same medium replacement rate as the SSB and static cultures with 100% of the medium replaced every three days. Fresh medium was constantly added at an average rate of 0.046 ml/min (200 ml/3 days) and removed at the same average rate to maintain a consistent culture volume. Later experiments utilized a variable feed rate to maintain nutrient levels based on the rate of cell usage.

### Continuous Feeding System

The continuous feeding system design was based on systems described in literature [16]–[19], [34]–[36]. Briefly, the system consists of five primary components: a media reservoir, peristaltic pump, stirred bioreactor, waste reservoir, and custom designed tubing/sampling set. The medium reservoir consisted of a 1 L glass bottle (Corning), the waste reservoir was a 2 L glass bottle (Corning), and the stirred bioreactor was a 250 ml volume glass reactor (Corning). A Masterflex digital peristaltic pump with an 8 channel pump head (Cole Parmer, Vernon Hills, IL) was used to control medium exchange. Reservoir and bioreactor lids were manufactured from hard plastic (Delran, Dupont, Wilmington, DE) with stainless steel pipe pass-through ports providing ventilation through sterile filters, and allowing for medium transfer between reservoirs and bioreactor. The bioreactor lid contained additional pass-through ports for optional instrumentation and monitoring probes (eg. oxygen monitor). The bioreactor lid also contained a novel outflow tube (OT) fabricated from porous glass aeration tubes that had an average pore size range of 40 µm to 60 µm, and a pore density of 40%. The OT pore size was chosen to remove only medium and cell debris, leaving cell aggregates in culture.

Custom autoclavable perfusion tubing sets were assembled from polyvinylidene fluoride (PVDF) tubing connectors and PharMed BPT Tubing (PharMedCorp, Westlake, OH). The tubing set consisted of three parts: a feed line, a waste line, and a sample line. The feed line was assembled using L/S 14 tubing for the primary lengths with a disposable L/S 13 section spliced in the middle as a replaceable “pump section.” This line connected to the fresh medium reservoir stored inside a refrigerator to reduce soluble factor degradation, passed through the pump head, and terminated at the feed port of the stirred bioreactor inside a NuAire (Princeton, MN) cell culture incubator. The second line for bioreactor waste removal was similar to the feed line but consisted of L/S 16 tubing for its primary lengths, and L/S 14 tubing for the replaceable “pump section.” The tubing diameters used for the waste line were larger to allow removal of medium from the bioreactor at the same rate as fed conditions to avoid significant culture volume changes, and to avoid the possibility of media overflows. The same tubing diameters could not be used to accomplish this task because any small variability in the tubing quality could result in a removal rate that was slower than the feed rate. Effectively, the waste line was pumping at a higher speed than the feed line because of the larger diameter tubing, and the volume in the reactor was controlled by setting the OT to the appropriate level.

The system functions by continuously pumping medium into the bioreactor, resulting in a rise in the level of medium in the bioreactor until reaching the height of the OT. The medium is removed through the OT, leaving the cell spheroids in culture, until the medium level falls below the bottom of the OT. This method of volume control with continuous feeding is used in many large-scale culture applications used to generate cell products [34], [35]. The system is designed to be simple without the need for specialized sensors and automation systems to control pump speeds. The medium level in the bioreactors fluctuates slightly because the surface tension of the media allows the outflow tube to remove medium briefly from the bioreactor after the average medium level is below the bottom of the tube. This small variability in culture volume does not exceed 6% of the total culture volume, and did not appear to adversely affect the spheroids during culture. To avoid vapor lock or pressure build up inside the sealed vessels, sterile vents were added to allow sterile air exchange. This was also important for allowing gas exchange between the incubator (5% CO_2_) environment and the bioreactor, which is necessary to maintain the correct pH.

The final component of the tubing set was a sample collection assembly. Briefly, it consisted of three short (∼6 cm) L/S 14 tubing lengths connected together with a T-type PVDF connector, and two small hose clamps on two of the tubing lengths. One of the clamped lengths contained a sterile gas filter for gas venting, and the other was used for connection to a sterile sampling syringe. The third end was connected to a stainless steel sample connector attached on the lid of the bioreactor. This entire assembly was autoclavable and connected to the bioreactor for the duration of the experiment. The assembly was used to collect a sterile sample from the bioreactor as needed without disturbing the continuous feed process.

### Spheroid Settling Rate Measurements

In order to ensure cell clusters were not being removed by the continuous feeding system, the settling rate of β-TC6 spheroids were measured by observing their descent in a large diameter plastic pipette. β-TC6 cells were cultured in SSB bioreactors to form spheroids as described above. After 3 days of culture, 30 ml samples of the spheroid suspension were gently pipetted to distribute evenly in suspension and placed in a large diameter 25 ml pipette, and the time for all of the spheroids to settle 5 cm was recorded. This was repeated three times for each of the three separate samples, and the data was averaged and used to determine the linear settling rate of the cells.

### Cell Counts, Viability and Glucose Concentration Measurements

Samples were collected from each bioreactor every three days before a medium change. For static and SSB culture methods all cells were collected as described above, and samples were taken from the cell suspension for cell counts and medium glucose measurements. Samples were collected using a wide orifice pipette to carefully suspend the spheroids and remove a representative sample, which consisted of no more than 2% of the total culture. Continuously fed SSB cultures were sampled using a specialized sampling port. For all culture methods, cells were dissociated by incubation for 2 minutes in trypsin-EDTA (0.25% (w/v) Trypsin- 0.53 mM EDTA, Invitrogen), and counted in triplicate using a standard hemocytometer with trypan blue staining [37]. Cell viability was calculated by recording live (unstained) cells, and dead (stained) cells independently (viability % = live cells/total cells). Cell growth rates are reported as a fold expansion percentage, calculated by dividing the live cell count after 21 days of culture to the original cell count at the beginning of the culture (Day 0). Glucose levels were measured in triplicate using a blood glucose meter (One Touch Ultra, Johnson & Johnson, New Brunswick, New Jersey), and single use test-strips (one touch ultra-strips). Both the fresh medium and waste medium were tested for glucose concentration, and measurements lower than 20 mg/dL (detectable threshold of the meter), although undetectable, were plotted as 20 mg/dL.

### Statistics

All data are reported as the mean +/− the standard error of the mean for at least three discrete measurements. Cell counts and glucose measurements were done in triplicate, and the two tailed un-paired student-t test was used to compare conditions. A p value<0.05 was considered significantly different.

## Results

### β-TC6 Cell Expansion in Static and SSB Cultures

**Figure 1:**
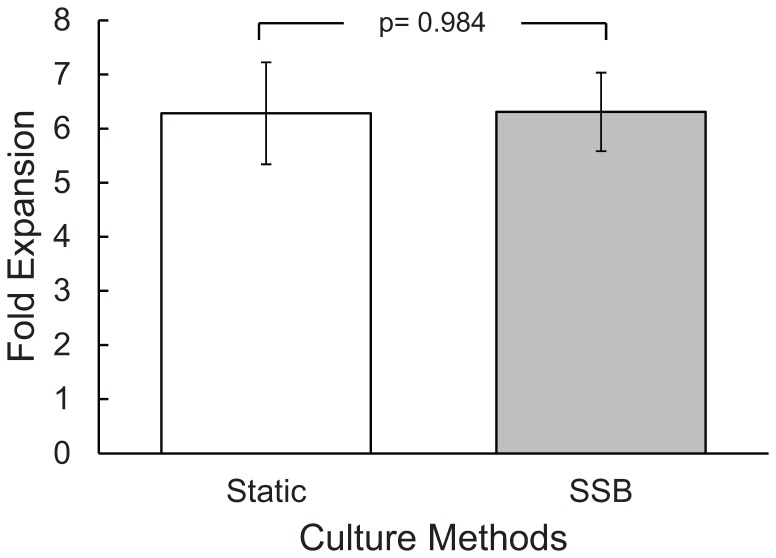
Stirred suspension bioreactors offered no growth improvement compared to static cultures. Fold expansion of β-TC6 spheroids after twenty one days compared stirred suspension bioreactor to static culture using standard high glucose medium.

In order to determine whether clusters of β-TC6 cells could be scaled up simply by increasing culture volume in a stirred suspension bioreactor. Static and SSB cultures were compared directly for cell yield and growth rate over a 3 week culture period. Although the SSB culture offers benefits when compared with static culture, Figure 1 demonstrates that there was no improvement in cellular expansion when compared to static cultures. The batch fed method had the same fold expansion over 21 days, and offered no significant improvement in growth rate compared with the static culture method.

### Nutrient Fluctuations in Static and SSB Cultures

**Figure 2:**
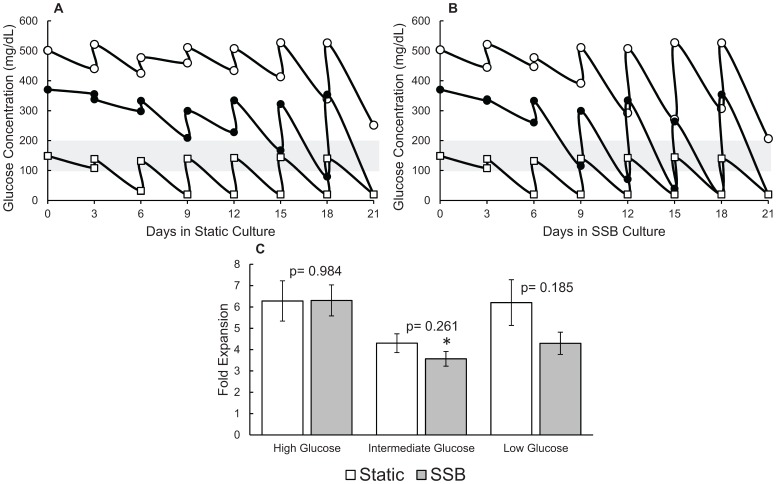
Fluctuations in medium nutrient levels contributed to limitations in cell expansion. Glucose measurements from β-TC6 cell spheroids cultured in (A) Static cultures, and (B) stirred suspension bioreactors using high (4.5 g/L), intermediate (2.75 g/L), and low (1.0 g/L) glucose medium, as depicted by their position on the Y axis. The physiological glucose range is indicated by the grey bar. Error bars for glucose measurements are too small to be visible on the scale shown (Standard Error ≤4% for all measurements). (C) No difference was seen comparing static to SSB cultures with any of the glucose levels. Comparison of expansion of β-TC6 spheroid cultures indicated that changing the glucose in the medium to achieve levels closer to the physiological range did not significantly improve cell expansion. (*indicates a p value of 0.027 compared with the same culture method using high glucose medium.) SSB: stirred suspension bioreactor.

As glucose is a key nutrient in cell culture, and glucose concentrations above normal are toxic to β cells over time in vivo [38], high and low levels of glucose were investigated as a limiting reagent for growth rate in static and SSB cultures with regular (3 day) medium changes. Static and SSB cultures were compared using standard medium with high glucose (4.5 g/L) as well as intermediate (2.75 g/L), and low (physiological) glucose (1.0 g/L) medium. Measurements were taken every 3 days, and it was found that static cultures experienced significant fluctuations in glucose levels (Figure 2a), which may limit cellular expansion and could diminish cellular viability and function. However, similar fluctuations were observed in the SSB cultures (Figure 2b), which may explain the similarities in cell expansion rates demonstrated in Figure 1 for static and SSB cultures. These results were consistent with the observation that no differences were observed in cell yields between static and SSB cultures (Figure 2c).

To determine whether the high levels of glucose observed when using standard medium or the fluctuations themselves were responsible for the limited cell expansion, cultures with different glucose concentrations were compared over the three week period. Culture medium with lower glucose concentrations could not maintain physiological glucose levels, and resulted in undetectable levels after 15 days of culture. Glucose measurements from β-TC6 spheroids using high (4.5 g/L) glucose medium resulted in super-physiological glucose levels for the duration of the 21 day culture period. Medium with intermediate (2.75 g/L), and low (1.0 g/L) glucose concentrations fluctuated between super-physiological and sub-physiological glucose concentrations, and resulted in levels below the detection threshold after 15 and 21 days of culture for SSB and static culture methods respectively. Despite maintaining glucose levels closer to the physiological range [39] in both culture methods by feeding with reduced glucose medium, Figure 2c illustrates that cell expansion was not improved, and was actually slightly reduced when compared to the standard (high glucose) cultures, although only the intermediate glucose SSB culture was significant (p = 0.027). The cell expansion was statistically similar between the static and SSB cultures at each glucose level. Thus, fluctuations in glucose levels are likely contributing to the limitation of cell expansion.

### Development of the Continuous Feeding (CF) Culture System

**Figure 3:**
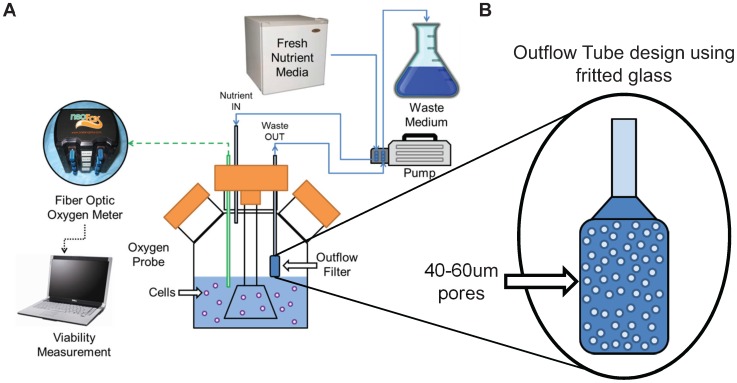
Design of continuous feed apparatus. (**A**) Schematic diagram of continuous feeding perfusion circuit with fresh medium, perfusion pump, outflow tube, and waste medium. Solid blue lines are medium connections, dashed green lines are fiber optic connections, and dotted black lines are electronic connections. (**B**) Exploded illustration of outflow tube showing small pores, which do not allow spheroids to pass through, but allow the removal of waste medium. The continuous feeding system was designed to help improve consistency of nutrient and supplement concentrations making β-TC6 cell culture parameters more stable and controlled.

To eliminate glucose fluctuations, a continuous feeding stirred suspension bioreactor system, shown in Figure 3a, was assembled, that continuously replenished medium in the culture system with no manual manipulation during the culture period [20], [21]. The perfusion circuit regulated the addition of refrigerated medium to the bioreactor while removing medium through the outflow tube at the same rate. The total culture volume was maintained by adjusting the depth of the outflow tube inside the bioreactor. The outflow tube illustrated in Figure 3b, allowed the removal of waste medium from the bioreactor without removing the cell spheroids using a fritted glass filter tube with small pores (40–60 um) that prevent the spheroids from being removed. The pore density was high (>40%) and the surface area was large, so that the linear flow velocity through each pore does not exceed the measured settling velocity of the spheroids. The linear flow velocity (V_P_) through a pore was calculated using Equation 1 below by dividing the maximum volumetric flow rate through the outflow tube (Q) divided by the total pore area calculated by multiplying the pore surface density (ρ_T_) by the bottom surface area of the outflow tube (A_T_).
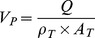
(1)


The average settling velocity for the spheroids measured after three days of SSB spheroid formation was observed to be 2.53±0.26 cm/min, which is more than ten times the calculated average linear medium velocity through a single pore (0.17 cm/min). This difference ensured that spheroids near the outflow tube were not pulled into the pores of the tube because they settled (due to gravity) much faster than the flow rate pulled them in. This design also ensured that the outflow tube would not become obstructed with spheroids.

### Continuous Feeding Eliminated Nutrient Fluctuations and Increased Cell Expansion

**Figure 4:**
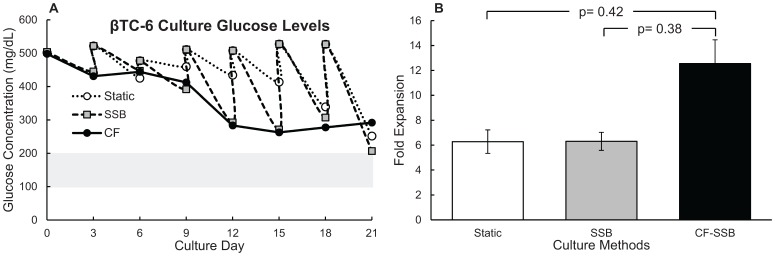
Continuously fed SSB eliminated glucose fluctuations and improved cell expansion. (**A**) Glucose measurements for β-TC6 spheroid culture medium using static, SSB, and CF-SSB culture methods and feeding with standard high glucose medium. The physiological glucose range is indicated by the grey bar. Error bars for glucose measurements are too small to be visible on the scale shown (Standard Error ≤4% for all measurements). (**B**) Fold Expansion of β-TC6 spheroids over 21 days of culture comparing static, SSB, and CF culture methods. SSB: stirred suspension bioreactor. CF: Continuously fed stirred suspension bioreactor.

Eliminating the nutrient fluctuations allowed greater cell expansion. The continuously fed culture was fed at a constant rate equivalent to the other culture methods, replacing 100% of the medium every three days. All cultures were fed with the same high glucose (∼500 mg/dL) medium. Figure 4a shows that continuous feeding at a constant rate eliminated the glucose fluctuations observed when using static or SSB culture methods, but resulted in a super-physiological concentration of glucose in the culture medium for the duration of the culture period. Eliminating these nutrient fluctuations resulted in the general improvement in cell expansion observed in Figure 4b. Spheroid size was compared on day 6 and day 21 for all conditions. The average sizes on day 6 were 0.20±0.01 mm, 0.17±0.01 mm, 0.18±0.01 mm for static, SSB, and CF-SSB respectively. The average sizes after 21 days of culture were 0.14±0.02 mm, 0.12±0.01 mm, and 0.28±0.04 mm for the same conditions. Average spheroid sizes were not significantly different between culture methods for all conditions after 6 days of culture (p>0.08), while the spheroids in the CF condition were significantly larger than either static or SSB cultures (p<0.02) after 21 days of culture. The larger size of the spheroids in the CF culture group is consistent with our cell count data, suggesting a higher growth rate for cells in the CF group. The average viability for all days and all culture conditions was 96.23±0.85%, and no differences were observed when comparing culture conditions or culture time points. This suggests that increased cell yields are primarily the result of increased growth rate, rather than reduced cell death following reduction of glucose fluctuations.

### Physiological Glucose Levels could not be Achieved by Feeding at a Constant Rate

**Figure 5:**
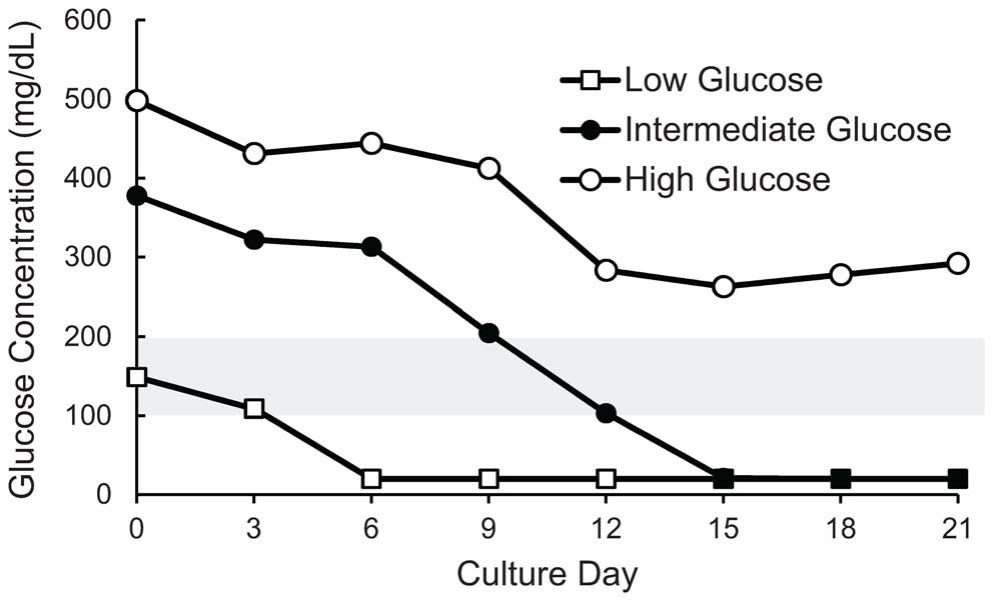
Continuous feeding at a constant rate cannot maintain a physiological glucose level. Culture medium glucose measurements from continuous fed cultures with high, intermediate, or low glucose medium during 21 days of expansion. The physiological glucose range is indicated by the grey bar.

Although the continuous feeding system eliminated the glucose fluctuations and improved the cell expansion, physiological levels could not be maintained using the recommended culture medium. In order to reduce the average glucose concentration in the culture, media with low or intermediate glucose levels were used. The continuous feeding system replaced medium in the culture at a rate of 100% medium change every three days. Figure 5 shows that using feed medium with decreased glucose concentration cannot maintain physiological levels for the duration of the 21 day culture period. Further, the glucose levels for the CF culture using the ATCC recommended high glucose culture medium stayed super-physiological for the entire 21 day expansion period, while the intermediate and low glucose medium cultures dropped to sub-physiological glucose levels by the 15^th^ day of culture, and this may explain the slightly reduced expansion for these conditions.

### Adjusting Feed Rate can Maintain Physiological Glucose Levels in SSB Culture

**Figure 6:**
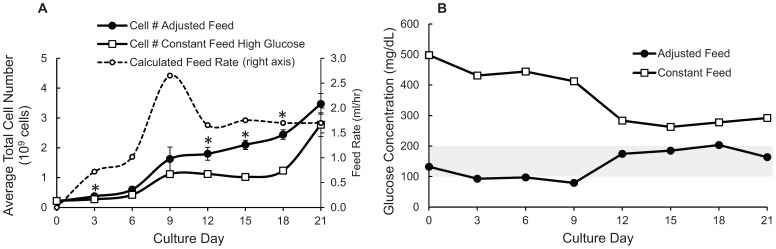
Adjusting culture medium feed rate regulates glucose concentrations and improves cell growth compared to continuous feeding at a constant rate. (**A**) Cell counts comparing constant feed rate to adjusted feed rate from the same cultures (*represents a p value <0.05 indicating a significant difference in culture expansion). (**B**) Average culture medium glucose levels from 21 day constant feeding, and adjusted feeding bioreactor cultures. The physiological glucose range is indicated by the grey bar. Error bars for glucose measurements are too small to be visible on the scale shown (Standard Error ≤4% for all measurements).

Continuously fed cultures with a constant feed rate were not able to maintain a physiological glucose level during the 21 day culture period. In order to maintain a more constant culture environment, an algorithm was developed to adjust the feed rate by incorporating data obtained from regular culture samples. The algorithm incorporated the predicted growth rate based on current cell counts, the predicted medium replacement rate based on historical glucose consumption measurements, and the adjusted feed rate based on current medium glucose measurements. Adjusting the feed rate based on this algorithm in a continuously fed SSB culture improved the cell expansion by 25% (Figure 6a) compared to the constant feeding method and the glucose level in the medium was maintained near physiological levels for the duration of the 21 day culture (Figure 6b).

### Predicted Linear Growth Rate

Equation 2 represents a linear approximation that was used to predict growth in culture. Previous spheroid culture data was fit, and a linear approximation most closely resembled the growth profile of the β-TC6 spheroids. This equation was used to predict the cell number every three days of culture. The equation calculates the predicted cell count (N_2_) by adding the cell count on the day of sampling (N_1_) to the linear growth rate approximated by fitting previous growth data (R_G_ = 1.26×10^8^ cells/day), which is multiplied by the culture period (t_2_–t_1_).
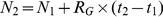
(2)


### Predicted Medium Replacement Rate

To predict the feed rate that was needed to replace the glucose consumed during culture, Equation 3 incorporated the predicted cell growth to calculate the glucose consumption during the culture period. The feed rate (R_F_) was calculated by assuming that it should equal the estimated glucose consumption of the entire culture and dividing by the glucose concentration in the feed medium (C_F_). This equation used the cellular glucose consumption rate (R_1_ = 1.25×10^10^ mg/cell/min), which was calculated by averaging the consumption rate empirically measured in previous SSB cultures over a 21 day culture period. The cellular consumption rate was multiplied by a weighted average of the cell number counted in a sample (N_1_) and the predicted cell number (N_2_) from Equation 2 to approximate the glucose consumption during the culture periods. The weighted average weights N_1_ two times over the predicted cell number in the average to increase the influence of the experimentally obtained data specific to each culture. This consumption rate was then divided by the glucose concentration (C_F_) in the feed medium (4.5 g/dL for these experiments) to obtain the predicted medium replacement rate needed to maintain a constant glucose concentration in culture.
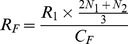
(3)


### Adjusted Feed Rate based on Glucose Levels

In order to incorporate actual glucose levels observed during culture, Equation 4 was added to provide a feedback control system. This adjusted the predicted culture medium replacement rate to account for the measured glucose levels in the culture medium on each sample day. The glucose adjusted feed rate (GAFR) is the rate that medium was actually replaced in the culture. This equation incorporated a unit-less engineering feedback control constant (X) ranging from 0–1 (value of 0.5 used for these experiments). It could be adjusted to change the amplitude of the feedback control with a smaller “X” causing less control, and larger “X” cause more control. C_1_ was the glucose concentration measured in the culture medium on the sample day, and C_D_ was the desired medium glucose concentration (100 mg/dL). The difference between C_1_ and C_D_ was divided by C_D_ to obtain a ratio describing the deviation from the desired glucose concentration, which was then multiplied by the control constant. The final value was subtracted from 1 to obtain the adjustment factor used to adjust the predicted medium replacement rate found in Equation 3 (R_F_). This adjustment decreased the feed rate when the glucose levels were higher than the desired concentration, and increased the feed rate when glucose levels were lower than the desired concentration. The effects of the feedback control are shown in Figure 6, where the calculated feed rate was plotted on the right hand axis. A large spike in the feed rate as calculated by the growth rate prediction part of the equation was observed on the 9^th^ day of culture, that was “corrected” downward by the feedback control on the 12^th^ day when the glucose levels in the medium were measured to be increasing above the desired concentration.
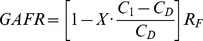
(4)


## Discussion

Cell therapies are the subject of intense research activity, and are becoming a realistic treatment option for many human diseases [40]–[43]. Many cell therapies will require the large-scale culture and monitoring of mammalian cells, as well as defined and validated culture conditions. Large-scale bioreactors provide an established approach from bio-pharmaceutical applications and culture of microorganisms that can be applied to the culture of mammalian cells with specific modifications to address this challenge. Mammalian cell culture techniques use static culture in disposable dishes for most research applications. Simply scaling up from static cultures to SSB methods still requires medium changes at regular intervals that result in dramatic changes in the cell culture environment. Cells are dosed with high concentrations of nutrients and growth factors at these batch-style medium changes to ensure survival between changes. During the culture intervals, cells experience steadily decreasing nutrient levels and increasing waste products. To address these issues in this study, glucose measurements were made to estimate degrading and consumed nutrients in the medium that spike immediately following medium change, and drop significantly over time, resulting in a constantly changing environment for the cells over the culture period. This study demonstrated that glucose levels fluctuated dramatically in the static and SSB culture of β-TC6 beta cell line, with 100% medium changes every 3 days. During cell expansion, when the cell density was greatest, glucose concentrations dropped as much as 275 mg/dL over three days. Although the glucose concentration never dropped to a physiological level during the 21 day culture period when culturing with DMEM high glucose medium (450 mg/dL) that is standard for the β-TC6 cell line, these fluctuations limited cell yield. The SSB medium glucose levels dropped at a similar rate with static cultures, and were nearly identical after 21 days of culture in static and SSB conditions.

Most cells experience environments in vivo that are more stable than cell culture conditions, with regulated levels of nutrients, and the constant processing and removal of cellular waste products. In this case, fluctuations in nutrient levels were sufficient to limit cell expansion. One strategy to improve the continuity of culture conditions is to increase the frequency of medium changes, decreasing nutrient level fluctuations and buildup of waste. Despite the improvements this approach would bring to nutrient and waste levels, for large-scale and long-term mammalian cell cultures, the time and cost associated with frequent batch-style medium changes make this approach impractical. The data presented in this study suggest that use of a stirred suspension bioreactor with growth adjusted continuous medium replacement can both maintain controlled culture conditions and improve culture outcomes without significant impact on cell viability. The increase in cell number and spheroid size, along with the consistent viability levels among culture groups suggest that CF culture conditions support increased growth rates in these cultures. Spheroids in static cultures were slightly larger than those in a SSB cultures, likely due to observed agglomerations under static conditions.

The adjusted feed algorithm used for these studies incorporated the linear growth rate of the cells in a spheroid, assumed a constant and average glucose consumption rate for all of the cells in culture, and included a feedback control system to account for the variability of individual cultures. A linear growth rate was used based on observed data from suspended β-TC6 cell spheroid cultures, but exponential growth, or more complex growth profiles could be used for various cell types. Glucose consumption and feeding can be generalized to a number of key nutrients in the medium. The glucose consumption used for these calculations were assumed to be constant and the same rate for each cell in the culture, but more complex glucose consumption relationships could be used in the same algorithm for cultures that contain more than one cell type or cell types that change during the culture period, such as differentiating stem cells. These techniques could also be used in conjunction with other advanced culture technologies including encapsulation, or micro-carrier surface cultures for adherent cells to improve cell growth rates. Any empirically determined glucose consumption profile specific to a cell type could be used to determine the needed medium replacement rate. In this study, the average glucose consumption rates observed in all conditions were similar to the rates observed in surface attached cultures suggesting that spheroid formation itself did not affect the glucose consumption of individual cells. The feedback adjustment system for this algorithm is a simple control that incorporates the scaled difference between the observed glucose levels and the desired glucose levels in the culture medium. More complex adjustment systems could be implemented to provide tighter culture control. The effects of this simple control system were apparent after nine days of culture (Figure 6a), when the predicted growth rate and glucose consumption rates were not able to maintain physiological levels on their own. The feedback control parameter was able to account for the increasing glucose concentrations in the culture, and adjust the predicted glucose replacement rate, decreasing it to account for the real-time observed glucose concentrations despite the increasing cell numbers. Furthermore, more complex cellular models tied to feedback control systems [44], [45] could be used to help regulate other culture parameters such as pH, dissolved oxygen concentration, and temperature. The adjusted feed system could be incorporated into a fully automated culture system [14], [22] and the feed rate could be adjusted based on more frequent sampling measurements which would further improve the control of nutrient levels in the medium. The automated system could control any culture parameters making mammalian cell cultures more consistent and production more reproducible.

Federal regulatory bodies have decided that mammalian cellular products for implantation or other cell therapy will be held to a similar standard as other transplant therapies. The industry is focused on developing and providing completely defined culture media [46]–[48] and working to meet these guidelines. Cell culture and manufacturing parameters will need to be controlled, monitored and recorded to ensure the efficacy and reproducibility of cellular products. As new products translate from the research lab into the clinic, the challenges of scale-up and production will be a significant barrier to implementation. As cell therapies are emerging as a treatment option for many human diseases, it is essential that culture parameters for cellular products are clearly defined, controlled, and monitored during the manufacturing process. Continuous medium replacement can reduce or eliminate fluctuations in critical nutrients observed in both static and stirred suspension bioreactor cultures. Incorporating an adjusted feed rate algorithm can further improve the control of culture conditions and can maintain near physiological nutrient levels for an extended culture period.

## Conclusions

The continuous feed system maintains glucose levels in the physiological range, when adjusted for cell growth and glucose consumption rate. Maintenance of glucose levels and removal of waste products continually supported cell expansion while maintaining viability. These systems could be adapted for expansion and differentiation of other cell types, including pluripotent stem cells, in addition to their utility for therapeutic mammalian cell spheroid culture.
